# The gene expression data of *Mycobacterium tuberculosis *based on Affymetrix gene chips provide insight into regulatory and hypothetical genes

**DOI:** 10.1186/1471-2180-7-37

**Published:** 2007-05-14

**Authors:** Li M Fu, Casey S Fu-Liu

**Affiliations:** 1 Pacific Tuberculosis and Cancer Research Organization, Irvine, California, USA

## Abstract

**Background:**

Tuberculosis remains a leading infectious disease with global public health threat. Its control and management have been complicated by multi-drug resistance and latent infection, which prompts scientists to find new and more effective drugs. With the completion of the genome sequence of the etiologic bacterium, *Mycobacterium tuberculosis*, it is now feasible to search for new drug targets by sieving through a large number of gene products and conduct genome-scale experiments based on microarray technology. However, the full potential of genome-wide microarray analysis in configuring interrelationships among all genes in *M. tuberculosis *has yet to be realized. To date, it is only possible to assign a function to 52% of proteins predicted in the genome.

**Results:**

We conducted a functional-genomics study using the high-resolution Affymetrix oligonucleotide GeneChip. Approximately one-half of the genes were found to be always expressed, including more than 100 predicted conserved hypotheticals, in the genome of *M. tuberculosis *during the log phase of in vitro growth. The gene expression profiles were analyzed and visualized through cluster analysis to epitomize the full details of genomic behavior. Broad patterns derived from genome-wide expression experiments in this study have provided insight into the interrelationships among genes in the basic cellular processes of *M. tuberculosis*.

**Conclusion:**

Our results have confirmed several known gene clusters in energy production, information pathways, and lipid metabolism, and also hinted at potential roles of hypothetical and regulatory proteins.

## Background

Knowledge about the genome sequence of *Mycobacterium tuberculosis *[1] has contributed to recent advancement in understanding the biology of this organism and its clinical relevance. Concurrent with this development, a high-throughput genome-wide gene expression analysis device in the form of microarrays has rapidly emerged as a seemingly indispensable tool for studying genomics in the modern era. These developments have brought about the revolutionary conception of new prophylactic and therapeutic interventions in the genomic perspective. Its significance should be clear, as tuberculosis is still causing millions of deaths in the world.

DNA microarrays have been applied to analyze *M. tuberculosis*. The first type of application focuses on genotyping, for example, species identification [2,3] and detection of drug-resistant mutants [4,5]. The second type of application seeks to explore altered gene expression and understand biological pathways in terms of up-regulated and down-regulated genes in certain conditions of interest, such as drug challenge [6], hypoxia [7], starvation [8], high temperature [9], and in vivo [10]. However, existing applications do not exploit the full potential of genome-scale microarray analysis in configuring interrelationships among all genes in *M. tuberculosis*. We pioneered the approach that applied the Affymetrix *M. tuberculosis *GeneChip to gene expression analysis. Previously, this GeneChip was used for applications related to genotyping.

Our study is aimed to explore the whole-genome behavior of *M. tuberculosis *during log-phase growth by conducting a bioinformatics analysis on genome-wide gene expression data generated from microarray hybridization. Our results enrich the current understanding of genome functions based on sequence analysis and functional studies of individual genes in such aspects as deduction of possible roles of conserved hypothetical and regulatory proteins.

## Results

### Active genes involved in growth

Research on *M. tuberculosis *has yet to answer the questions of how many and what genes are active during normal growth in a standard in vitro environment and how they are related to each other in a global genome-wide sense. To answer these questions, we adopted the Affymetrix GeneChip system, which, based on a specific oligonucleotide array format, could provide the absolute signal intensity in a single condition as well as the signal ratio between two conditions. Furthermore, its built-in statistical algorithm computes the so-called Detection *p*-value that determines the presence or absence of any given mRNA. It is this feature that we capitalize on to explore the genomic behavior of *M. tuberculosis*. A gene is active when it is expressed. Gene activity is measured by the expression level (i.e., abundance of corresponding mRNA) detected by microarray hybridization in this study.

We found that about one-half of the genes in *M. tuberculosis *genome participated in bacterial growth during the log (exponential) phase in standard broth culture. The average and standard deviation of expression signal intensity for each active gene would reflect its relative level of activity and the range of variation (Table 1). It was noted that the extent of gene expression did not necessarily correlated with essentiality; for example, among the ten most expressed genes, only two were essential genes as defined by high density mutagenesis [11].

### Functional genomic analysis

**Figure 1:**
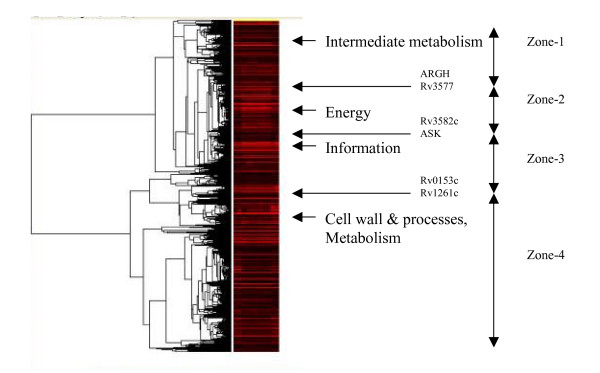
The gene expression map of genes involved in the log-phase growth of *Mycobacterium tuberculosis*. The map was generated by Eisen's cluster-analysis program called CLUSTER and viewed by the TREEVIEW program. Several clusters representing aggregations of functionally related genes are visible in the dendrogram (on the left of the image) showing how genes are grouped. The detailed image is available at our web site [see Additional file 2] annotated with gene names alongside of the image strip [see Additional file 3].

The microarray data of those genes involved in the in vitro growth of *M. tuberculosis *during log phase were analyzed through the hierarchical clustering algorithm of Eisen's Cluster program [12]. This cluster-analysis program allowed us to explore the internal structure of the data and derive useful information concerning the coordination and collaboration among the genes. A measure fundamental to clustering is that of similarity. We define similarity between genes by their correlation in terms of gene expression patterns across multiple samples. The dendrogram generated by the algorithm (Figure 1) was organized according to this measure and displayed in a way to optimize the similarity of adjacent elements (genes). In the dendrogram, more related genes were joined earlier, and several highly dense clusters with peaks spreading along were visible.

Based upon the above analysis, the dendrogram was partitioned, using a correlation threshold of 0.9, into a number of disjoint clusters related to functional classes known previously. Each cluster contained functionally related genes that might share similar functions or roles in physiology or metabolism and were likely subject to the same regulatory mechanism. For instance, one cluster was found to be similar to the FAS-II operon, and another cluster comprised largely of genes encoding ribosomal proteins (Table 2). Notice, however, genes of different classes may co-exist in the same cluster if they show correlation in their activity. This situation is exemplified by energy-dependent transportation across cell membrane, which necessitates the coupling between the gene class of cell processes and that of energy production.

The clustering results were further visualized through Eisen's TreeView program to generate a heat map where the brightness of the red color represented the intensity of gene expression (Figure 1). Based on the cluster analysis results and the observation of four conspicuous shining bands, the map was tentatively divided into four zones from the top to the bottom, called: zones 1, 2, 3, and 4, in the consecutive order. Each zone contained clusters of genes that were strongly correlated in their expression patterns and in that sense, functionally related. Each zone in the map was represented by genes that expressed most (i.e., the brightest in red), Thus, the map zones were labeled according to the functional class (genolist.pasteur.fr/TubercuList/) of their representative genes as "intermediate and lipid metabolism", "energy", "information", and "cell wall, cell processes, and metabolism", respectively.

The two most conspicuous clusters in the first zone of the gene expression map consisted of a cluster represented by genes in the functional category of intermediate metabolism, such as *cysH*, *ggtB*, and *nirA*, and the other cluster represented by genes involved in the FAS-II cycle [6], such as *accD*6, *kasA*, and *KasB*. The formation of the cluster with emphasis on the FAS-II cycle reflects the importance of this pathway in *M. tuberculosis *growth. As for intermediate metabolism, *M. tuberculosis *can metabolize many kinds of carbohydrates, hydrocarbons, alcohols, ketones and carboxylic acids [1]. This zone was adjacent to the energy zone, suggesting the close relationship between intermediate metabolism and energy production.

The most prominent genes in the second zone of the gene expression map were the ATP synthase gene complex, which produces ATP from ADP and is critical in energy metabolism. In addition, genes encoding enzymes involved in the respiratory chain, such as the *nuo *(NADH-ubiquinone oxidoreductase) gene complex, were clustered into this zone. In another study [13], ATP synthase and *nuo *gene complexes were found to be down-regulated together. Thus, they may be co-regulated.

The third zone of the gene expression map was represented by genes involved in the information pathways, e.g., *dnaQ *(DNA polymerase III), *recA *(recombinase), *rpoA *(DNA-directed RNA polymerase), and the 30S and 50S ribosome protein gene complexes (*rpsB*, *rpsC*, *rplB*, *rplC*, etc.). As these genes play a vital role in genetic information replication, transcription, and translation, their expression is essential for maintaining bacterial growth. We noticed that the gene Rv2258C (encoding a possible transcriptional regulatory protein) correlated well with a group of ribosomal protein genes. This result is related to a report that the production of ribosomes is increased through the transcriptional regulation of genes encoding ribosomal proteins during the growth phase of yeast [14].

The last zone of the gene expression map was represented by genes classified under the category of cell wall, cell processes, and metabolism. This category comprises membrane proteins and proteins involved in cell processes, including secreted and transmembrane proteins [15], as well as enzymes involved in intermediate metabolism. Genes and their protein derivatives located in this zone are related to cell wall synthesis, transportation of organic and inorganic substance across membrane, and immunological responses, such as *narK*2 (a nitrate/nitrite transporter), *fbpC *(mycolyl transferase), *hsp *(a protein induced by heat stress), *ald *(a secreted enzyme), *ctpF *(a metal cation transporter), *sodA *(superoxide dismutase, which destroys radicals), *ompA *(an outer membrane protein), *amt *(an ammonium-transport integral membrane protein), *furA *(a protein for ferric uptake regulation), *fbpB *(a protein in the antigen-85 complex), and genes in the lipoprotein family (*lpqS*, *lpqH*, *lppJ*, *lprF*, *lpqO *and *lppD*).

The most important feature of mycobacterial cell wall is the substantial amount (up to 60% of the total mass) of lipid components, particularly, the very long chain mycolic acids, which are combined with surface glycolipids to form a pseudolipid bilayer [16]. Since the cell wall synthesis involves lipid metabolism [6], the main cluster in zone 4 also contains some genes in this class, such as *fadE26 *(encoding acyl-CoA dehydrogenase) and *fadD16 *(encoding fatty-acid-CoA ligase).

Each zone was tentatively named according to the most expressed genes it contained. The semantics of each zone would further depend on whether each zone represents a concentration of genes in the same functional categories and that concentration is consistent with the function classification of the most expressed genes in that zone. So we further analyzed the percentage distribution of functional classes for the genes in the major clusters of each zone on the gene expression map (Table 3). The functional classification of a gene is based on current knowledge in the field and can be accessed from the TubercuList Server (genolist.pasteur.fr/TubercuList/). Our analysis indicated that each zone contained clusters of genes of similar functions and the most expressed genes were in the dominant function class, suggesting that functional semantics can be assigned to clusters visible from the gene expression map. Zone-1 featured genes in the functional classes of metabolism (intermediate and lipid). In zone-2, there was only a slight dominance of genes in the class of metabolism and respiration over the class of cell wall and processes, but notably, it contained a big chuck of energy-related genes involved in ATP synthesis and electron transport. Zone-3 was characterized by genes in the information category. Zone-4 had the broadest distribution on the map and a wide functional coverage, and was thus least characteristic among the four zones.

There were about a dozen of regulatory genes associated with the major functional clusters identified in this study (Table 4). Their significance is reflected by their possible roles reported in other studies. These genes likely regulate other genes in the same cluster at the transcriptional level and deserve to be examined in more details. Regulatory genes associated with small clusters are considered less important and hence not examined here.

The Rv2989 gene was associated with the cluster characterized by energy metabolism and respiration in our data. The association relation appears to be consistent with reports that this gene is up-regulated at high temperatures [9] and down-regulated after starvation [8].

Rv3334 was up-regulated at high temperatures [9] as well as after starvation [8]. This gene is probably in the MerR (mercury resistance) family and its protein is similar to many regulatory proteins in sequence.

Rv0081 can be induced by hypoxia [7]. Its presence in growing bacterial cells is called into question. However, this gene is only weakly expressed in the present study, and likely to be up-regulated if oxygen is depleted.

The *whiB4 *gene encodes a protein homologous to a Streptomyces sporulation factor [17], and the gene is up-regulated after starvation [8], suggesting a possible link between starvation and sporulation. In addition, the association of *whiB4 *with the class of cell wall and cell processes makes sense from the point that sporulation could potentially involve cell membrane.

Rv0653c is probably in the TetR family. Its significance is reflected by the fact that proteins in this family are involved in the transcriptional control of multi-drug efflux pumps and pathogenicity [18]. Another regulatory gene, Rv0165c, is probably in the GntR family. In *E. coli*, GntR regulates gluconate uptake and catabolism as a repressor [19]. Both Rv0653c and Rv0165c are apparently involved in a membrane-associated cellular process. Another gene worth attention is Rv1931c, which regulates genes important for virulence of *M. tuberculosis *[20], possibly via a cellular process that translates extracellular stimuli into a transcriptional signal.

## Discussion

The availability of the complete genome sequence of *Mycobacterium tuberculosis *[1] combined with rapidly emerging microarray technology [21] has catalyzed the process of understanding the bacterial biology and pathogenicity and expedited the development of new diagnostics and therapeutics for tuberculosis. The microarray approach has enabled high-throughput gene expression analysis on a genomic scale in a field known as functional genomics [22]. In particular, elucidation of functional relationships among genes based on genome-wide gene expression data from DNA microarray hybridization has been successfully demonstrated for eukaryotes, notably yeast [12,23], but it has not been done for *M. tuberculosis*. To date, the functional classification of genes in *M. tuberculosis *is mainly based on the biological study of individual genes as well as sequence analysis and comparison with homologous genes in other bacteria. In this study, we provide a comprehensive analysis that addresses this issue from the perspective of functional genomics.

In the application to *M. tuberculosis*, DNA microarrays have been used for comparing species, detecting drug-resistant mutants, and studying biological behavior under various conditions. In general, there are two computational paradigms for microarray data analysis. The first paradigm is to identify genes differentially expressed across two conditions; the second paradigm is to identify genes expressed in a coordinated manner that share common roles in cellular physiology or metabolism. Most of the applications for *M. tuberculosis *to date are based on the first paradigm, whereas our study described here is based on the second paradigm.

In this study, active genes were identified by means of the Affymetrix GeneChip. As its unique feature, the Affymetrix system uses multiple oligonucleotide probes for implementing each gene sequence to be interrogated. Furthermore, the system is capable of analyzing the presence or absence of each mRNA. In contrast to the cDNA microarray system that is focused on differential gene expression across two conditions, the Affymetrix system can calculate gene expression in a single condition and compare gene expression across multiple conditions. In this way, the Affymetrix system is more flexible and informative. The flexibility can be attributed to the use of PM/MM probes, instead of two explicitly defined external conditions, for implementing the test/control mechanism in microarray hybridization.

Our method for analysis of in vitro genomic activity of *M. tuberculosis *can be extended to study functional genomics in vivo. Understanding what genes are switched on or off between in vitro and in vivo conditions would shed light on issues, such as how biological adaptation leads to bacterial latency, why there is discrepancy between laboratory sensitivity and clinical efficacy, and so on. Thus, the functional-genomics data obtained in this work can serve as a reference for interpreting data generated in other contexts. Our genomic analysis was based on multiple RNA samples extracted during log-phase growth in contrast to other coordinated gene expression analyses based on samples collected in a time course or under different conditions. Our experiment design is justified, given the fact that the expression level of any gene has considerable fluctuation from time to time (Table 1) in log-phase growth, as evidenced from the observation that the standard deviation of a gene expression was sometimes greater than 50% of its mean across samples. Variation in gene expression across different time settings enabled the correlation among different genes to be analyzed. The validity of our experiments is supported by reconfirmation of several known growth-related gene clusters. However, our approach is not applicable to samples collected during stationary phase, when little variation in gene expression is expected across samples.

The in vitro broth culture condition has often been used as the reference condition to study the gene expression of *M. tuberculosis *in other conditions, such as hypoxia and starvation. However, the present study is the first to explore the functional genomics of this organism grown in log-phase culture. Bacterial growth can be divided into four different phases: lag phase, exponential or log phase, stationary phase, and death phase. It is the log phase that we focused our study on. During this phase, high growth activity is evidenced by our data showing that about half of the genes in the genome were expressed. In contrast, many genes in *M. tuberculosis *are repressed during the stationary phase, a condition similar to but milder than the non-replicating state of tubercle bacilli in an anaerobic condition [24]. In particular, the dormancy regulatory gene, *dosR *is weakly induced during the stationary phase while strongly induced in an anaerobic non-replicating state. An interesting finding based on our work is that *dosR *is always moderately expressed even in the log phase, suggesting its possible housekeeping role. Our data further showed that an important gene, *acr *(*hspX*), which is induced under hypoxia [7] and starvation [8], was always expressed in the log phase. Global gene expression profiling analysis of *M. tuberculosis *in mouse [25] and human tissue [10] indicated that lipid metabolism was critical for the bacilli to survive in the host environment. In these conditions, isocitrate lyase (ICL), an enzyme of the glyoxylate shunt (a pathway alternative to the tricarboxylic acid cycle) and related to mycobacterial persistence in macrophages [26], is up-regulated. It is consistent with our finding that *icl *is weakly expressed or absent in the log-phase culture.

The extent to which gene expression profiles across a set of independently collected samples suffice to separate genes into functional clusters in consistency with prior knowledge is attributable to the rigorous statistical model built in the Affymetrix system. Several familiar gene groups with clear designated functions, such as electron transport, protein synthesis and type II fatty acid synthesis, were observed in the data, offering credence to our analysis. However, genes associated with different functional classes would be placed in the same cluster if they appear to co-express. This implies that genes can share commons roles while differing in their functions, as illustrated by the earlier example of energy-dependent transportation across the cell membrane. In fact, comparing gene clusters based on gene expression with gene classes based on sequence analysis would offer new opportunities for re-defining interrelationships among genes in the genome.

Gene clusters built out of expression profiles can be configured as functional linkage networks among genes but these clusters do not correspond directly to protein networks [10] constructed using a combination of Rosetta stone, phylogenetic profile, conserved gene neighbor, and operon computational methods. Genes, which share similar biological functions, may operate at different stages of the cell cycle or become active under different conditions, and hence have different expression profiles [10]. However, some gene families, in particular those encoding ribosome proteins are closely linked in both gene-expression and protein networks, as seen in our data.

There remained 1051 so-called conserved hypothetical genes/proteins annotated in the genome sequence of *M. tuberculosis *[15], among which many were found to be active in our analysis, attesting to the predictive power of the genome annotation program [1]. More importantly, this finding suggests that many hypothetical proteins actively participate in the growth process. Unfortunately, the functions of these hypotheticals remain uncharacterized, reflecting the limitation of the sequence-based approach to genome annotation. Microarray-based functional-genomics offers a fundamentally different approach to gene annotation, in which the roles of uncharacterized genes may be hypothesized if they co-express with known genes (Table 5).

Organized according to correlations in gene expression across samples, the gene expression image created by Eisen's Cluster and TreeView programs (Figure 1) enabled us to visualize four transcriptional profiles, which, named according to the functional classes of the dominant genes in that region and put in a linear order over the image, were "intermediate and lipid metabolism", "energy and respiration", "information pathways", and "cell wall, cell processes and metabolism". The dendrogram was constructed and displayed so that similar clusters were likely to be located in proximate nodes. In the present application, as the similarity measure is based on the correlation in gene expression, physical distance on the tree reflects the degree of correlation among gene clusters, even with no guarantee of their optimal linear ordering in the tree [12]. As the expression of the genetic code lies at the heart of all physiological processes and metabolisms, it is logical that the information gene cluster functionally correlates with other gene clusters, a view supported by the observation that it was situated around the center of the image.

As it is now, there are more than 100 transcriptional regulatory genes in *M. tuberculosis *genome [1,15]. However, only a fraction of them have been experimentally studied in detail for their functions. A recent survey shows that regulatory proteins account for 9 (20%) out of 45 virulence factors identified in *M. tuberculosis *[27]. Unraveling the gene regulatory network would allow us to understand both physiological and virulence mechanisms and to develop novel drugs that work at the level of gene regulation. Since elucidating the roles of these genes and their clinical relevance is always time-consuming; it is practically necessary to set up priority for them. A reasonable assumption is that a regulatory gene regulates some other genes in the same functional cluster with a high probability. Under this assumption, we have identified several potentially important transcriptional regulatory genes involved in major biological pathways (Table 4). Their significance has been indicated by analysis based on the literature. Further biological investigation on these genes is warranted in the future work.

All the microarray data and supplementary materials produced in this study are posted at our web site [see Additional file 1].

## Conclusion

Genes involved in the in vitro log-phase growth of *M. tuberculosis *have been identified. The gene expression map (Figure 1) represents broad patters of functional concordance of closely related genes, but more importantly, it summarizes the coordinated cellular activities associated with the growth process on the genomic level. As it is today, hundreds of genes in the genome are annotated as conserved hypotheticals without clearly specified functions. Our data have shown that more than 100 such hypotheticals were actually expressed in the cell medium, and their biological roles can be suggested by their correlation with other known genes. In addition, the roles of most transcriptional regulatory genes predicted in the genome remain to be elucidated. In this study, we have discovered several regulatory genes that may exert regulatory influence on the growth of *M. tuberculosis*, and their roles may be inferred by what functional clusters they join. The data and information generated here provide an integrated genomic view about gene functions and interrelationships in *M. tuberculosis*, and can be incorporated in new experiments for research in tuberculosis. This study has not only transcriptionally validated several known gene clusters but also provided insight into a host of unknown hypothetical and regulatory genes.

## Methods

### Bacterial culture of *M. tuberculosis*

*M. tuberculosis *strain H37Rv was obtained from the culture collection of the Mycobacteriology Laboratory Branch, Centers for Disease Control and Prevention at Atlanta. A portion of a recently frozen stock was inoculated into 5 ml of complete Middlebrook 7H9 broth (7H9) supplemented with 10% albumin-dextrose-catalase v/v (Difco Laboratories, Detroit, MI) and 0.05% Tween 80 v/v (Sigma, St. Louis, MO) and incubated at 37°C for 5 days. Then the culture was transferred into 50 ml of 7H9 media, incubated at 37°C with 50 rpm shaking, and grown to log phase (0.35 OD_600_). The cells were harvested by centrifugation for RNA preparation.

### RNA isolation

Bacterial lysis and RNA isolation were performed following the procedure of [28] at the CDC lab (Atlanta) during log-phase growth. Briefly, cultures were mixed with an equal volume of RNALater™ (Ambion, Austin, TX) and the bacteria harvested by centrifugation (1 min, 25000g, 8°C) and transferred to Fast Prep tubes (Bio 101, Vista, CA) containing Trizol (Life Technologies, Gaithersburg, MD). Mycobacteria were mechanically disrupted in a Fast Prep apparatus (Bio 101). The aqueous phase was recovered, treated with Cleanascite (CPG, Lincoln Park, NJ), and extracted with chloroform-isoamyl alcohol (24:1 v/v). Nucleic acids were ethanol precipitated. DNaseI (Ambion) treatment to digest contaminating DNA was performed in the presence of Prime RNase inhibitor (5'-3', Boulder, CO). The RNA sample was precipitated and washed in ethanol, and redissolved to make a final concentration of 1 mg/ml. The purity of RNA was estimated by the ratio of the readings at 260 nm and 280 nm (A260/A280) in the UV. 20 ul RNA samples were sent to the UCI DNA core and further checked through a quality and quantity test based on electrophoresis before microarray hybridization.

### Microarray hybridization and analysis

In this study, we used the anti-sense Affymetrix *M. tuberculosis *genome array (GeneChip). The probe selection was based on the genome sequence of *M. tuberculosis *H37Rv [1]. Each annotated ORF (Open Reading Frame) or IG (Intergenic Region) was interrogated with oligonucleotide probe pairs. The gene chip represented all 3924 ORFs and 738 intergenic regions of H37Rv. Twenty 25-mer probes were selected within each ORF or IG. These probes are called PM (Perfect-Match) probes. The sequence of each PM probe is perturbed with a single substitution at the middle base. They are called MM (Mismatch) probes. A PM probe and its respective MM probe constitute a probe pair. The MM probe serves as a negative control for the PM probe in hybridization.

Microarray hybridization followed the Affymetrix protocol. In brief, the assay utilized reverse transcriptase and random hexamer primers to produce DNA complementary to the RNA. The cDNA products were then fragmented by DNAase I and labeled with terminal transferase and biotinylated GeneChip DNA Labeling Reagent at the 3' terminal.

Each RNA sample was hybridized with one gene array to produce the expression data of all genes on the array. We performed eleven independent bacterial cultures and RNA extractions at different times, and collected eleven sets of microarray data for this study. A global normalization scheme is applied so that each array's median value is adjusted to a predefine value (500).

### Bioinformatics analysis

The gene expression data were analyzed by the program GCOS (GeneChip Operating Software) version 1.4. In the program, the Detection algorithm determines whether a measured transcript is detected (P Call) or not detected (A Call) on a single array according to the Detection *p*-value that is computed by applying the one-sided Wilcoxon's signed rank test to test the Discrimination scores (R) against a predefined adjustable threshold τ. The Discrimination score calculated for each probe pair is a function of the PM intensity (PMI) and the MM intensity (MMI), as given by

R = (PMI - MMI)/(PMI + MMI)

The parameter τ controls the sensitivity and specificity of the analysis, and was set to a typical value of 0.015, and the Detection *p*-value cutoffs, α_1 _and α_2_, set to their typical values, 0.04 and 0.06, respectively, according to the Affymetrix system.

In this study, a gene was determined to be always (usually) active if the derived mRNA was present (P-call) in more than 90% (50%) of the RNA samples with a Detection *p*-value < 0.001. The gene-expression data were further analyzed using Eisen's Cluster and TreeView programs [12]. The whole-genome gene expression map was produced by the hierarchical clustering algorithm based on the average-linkage method in the program with the similarity measure defined by Pearson's correlation coefficient.

## Authors' contributions

L. Fu developed the method, conducted the experiments, and performed data analysis. C. Fu-Liu interpreted the data. Both authors drafted, read, and approved the manuscript.

## Supplementary Material

Additional File 1 Microarray data. The microarray data and supplementary materials produced in this study. Click here for file

Additional File 2 Gene expression map (detailed full image). *M. tuberculosis *during log-phase growth. Click here for file

Additional File 3 Gene names alongside the gene expression map. in the order as they appear in the map. Click here for file

Additional File 4 Active genes. *M. tuberculosis *during log-phase growth. Click here for file

Additional File 5 Gene zones in the gene expression map. *M. tuberculosis *during log-phase growth. Click here for file

## Figures and Tables

**Table 1:** The top 100 most expressed genes of *M. tuberculosis *in log-phase growth. For other active genes, refer to Additional file 4.

ORF	Gene	Mean	S.D.	ORF	Gene	Mean	S.D.
Rv1641	*infC*	11397	1612	Rv0709	*rpmC*	4436	859
Rv1872c	*lldD2*	10798	1943	Rv0170	*mce1B*	4383	723
Rv1398c	-	10543	2353	Rv3219	*whiB1*	4380	467
Rv1038c	*esxJ*	10524	1911	Rv1094	*desA2*	4331	359
Rv1037c	*esxI*	9677	2110	Rv3053c	*nrdH*	4315	430
Rv3874	*esxB*	9539	2143	Rv2007c	*fdxA*	4217	1604
Rv3614c	-	8992	3003	Rv1388	*mihF*	4191	611
Rv2031c	*hspX*	8926	2861	Rv0704	*rplB*	4140	917
Rv3615c	-	8718	2869	Rv1980c	*mpt64*	4063	1067
Rv3648c	*cspA*	8362	1199	Rv3459c	*rpsK*	4044	393
Rv3131	-	8102	3991	Rv0682	*rpsL*	4034	393
Rv2348c	-	7796	2033	Rv0715	*rplX*	4004	773
Rv3407	-	7792	1011	Rv3130c	-	3991	1822
Rv3616c	-	7388	2263	Rv2442c	*rplU*	3988	432
Rv3583c	-	7366	851	Rv0718	*rpsH*	3982	799
Rv0288	*esxH*	7332	1684	Rv3412	-	3952	331
Rv3841	*bfrB*	7057	1518	Rv1306	*atpF*	3857	609
Rv1871c	-	7039	840	Rv0655	*mkl*	3727	408
Rv3408	-	6953	884	Rv1174c	*TB8.4*	3721	321
Rv1397c	-	6931	1091	Rv0708	*rplP*	3658	825
Rv3804c	*fbpA*	6875	410	Rv0440	*groEL2*	3650	935
Rv0703	*rplW*	6378	651	Rv0668	*rpoC*	3637	661
Rv3461c	*rpmJ*	6349	379	Rv3849	-	3615	650
Rv1298	*rpmE*	6160	765	Rv1211	-	3606	386
Rv3418c	*groES*	6017	1376	Rv2204c	-	3605	594
Rv1177	*fdxC*	6004	569	Rv1310	*atpD*	3604	694
Rv0685	*tuF*	5895	438	Rv3281	-	3579	602
Rv1297	*rhO*	5609	950	Rv3051c	*nrdE*	3490	405
Rv3460c	*rpsM*	5602	552	Rv0009	*ppiA*	3479	519
Rv0824c	*desA1*	5569	698	Rv2161c	-	3452	934
Rv2094c	*tatA*	5552	866	Rv1308	*atpA*	3439	467
Rv3462c	*infA*	5324	291	Rv0167	*yrbE1A*	3439	620
Rv0700	*rpsJ*	5315	464	Rv3457c	*rpoA*	3396	841
Rv1072	-	5244	450	Rv0174	*mce1F*	3393	604
Rv0144	-	5177	653	Rv0641	*rplA*	3376	464
Rv0287	*esxG*	5135	1345	Rv2457c	*clpX*	3371	337
Rv2986c	*hupB*	5127	953	Rv1109c	-	3366	335
Rv2137c	-	5070	962	Rv2785c	*rpsO*	3361	365
Rv3127	-	4963	1788	Rv3052c	*nrdI*	3356	295
Rv2840c	-	4959	277	Rv0710	*rpsQ*	3323	715
Rv3679	-	4952	995	Rv0639	*nusG*	3320	749
Rv0706	*rplV*	4943	950	Rv0569	-	3283	1757
Rv1738	-	4921	2210	Rv1827	*cfp17*	3214	212
Rv0702	*rplD*	4868	415	Rv2159c	-	3197	832
Rv0701	*rplC*	4822	429	Rv0640	*rplK*	3169	256
Rv1642	*rpmI*	4816	562	Rv2392	*cysH*	3147	663
Rv2244	*acpM*	4650	1053	Rv2196	*qcrB*	3137	578
Rv1884c	*rpfC*	4642	615	Rv2391	*nirA*	3110	822
Rv1305	*atpE*	4488	679	Rv0289	-	3088	583
Rv0705	*rpsS*	4476	908	Rv3153	*nuoI*	3066	747

S.D.: Standard deviation.

**Table 2:** 

**Zone**	**Gene Clusters**	**Representative Gene Class**
1	(CYSH Rv1478 GGTB NIRA Rv2425C ACCD4 Rv3541C Rv0175 Rv2393 MURX Rv3701C) (PNTAB KASA HEMC LPQI ACCD6 KASB Rv2052C Rv1728C ARGH)	Intermediate and lipid metabolism
2	(DRRB Rv1251C EFPA Rv2054 MURC Rv1632C Rv2395 Rv1378C QCRA CTAE RHO PARB Rv3321C ATPH Rv3921C ATPD Rv3805C Rv2901C PARA Rv2949C CTAC Rv1576C ATPB Rv0546C Rv2781C Rv0526 Rv3104C POLA PYRH Rv1870C Rv1711 Rv3672C Rv0514 DAPF Rv2554C Rv1869C NUOE NUOC Rv1178 Rv0528 Rv1481 Rv2791C Rv2610C Rv3856C Rv1565C Rv3212 Rv1043C TSNR Rv1324 PGK Rv0525 RUVC KSGA Rv2989 LPRE PURK Rv0412C RODA Rv3725 HEME Rv1339 Rv1797 NRP UREC Rv2852C Rv3781 TRPA Rv2956 TRPB Rv2808 Rv2128 Rv1695 NUOI Rv1312 NUOL NUOD Rv3806C Rv2759C Rv2966C FOLK Rv2879C Rv1780 Rv1271C Rv3693 PLCB DRRC Rv2600 NUOM NUOH Rv0875C Rv3220C Rv3885C MMPL7 Rv2475C LTP1 Rv0236C NUOJ NUOB NUON MMPL9 Rv2752C Rv0177 Rv0176 HEMB PRCA Rv2553C Rv2367C RPLT GID CMK Rv3122 EMBB Rv1303 Rv1907C Rv2792C)	Energy production and respiration
3	(FTSZ WAG31 Rv0902C HEMK NARL Rv2147C Rv3267 Rv1477 LPPW SIGC Rv2864C RECA Rv2826C Rv1697 LEUC LEUD Rv3909 DNAQ Rv1465 Rv0486 Rv3910 PIRG Rv2574 Rv2360C Rv3816C AROB Rv2827C FTSK Rv3587C FOLE FTSQ Rv3647C Rv3376 RHLE) (LEUA AMIC RPOA RPSE RPLF RPSH RPLX Rv0203 RPLJ RPLR RPLE RPLB RPLC NUSG) (RPSS ALR RPSC RPLV PNTB RPLP RPMC Rv2125 PRFA Rv1546 Rv3278C Rv1099C RPLO TPI Rv0299 Rv3677C Rv2258C) (RIMM Rv1258C RPLL Rv2908C RPLI Rv2822C PCKA Rv1073 Rv0636 Rv0637 CYSA2 Rv0277C RPSP RPSB LPPU HTPG RPLM RPSI SSEC2 Rv0057 SSB ASPC)	Information pathways (replication, transcription, and translation)
4	(SIGE Rv0516C Rv0846C Rv0991C Rv3334 Rv2628 Rv2020C Rv0968 Rv2517C NARK2 FBPC LPQS Rv2662 Rv1772 Rv0967 Rv0465C Rv1813C Rv2016 HSP Rv1847 Rv0190 Rv1774 RPST ALD Rv0080 Rv2699C Rv2629 Rv0571C Rv2623 Rv0572C Rv2005C CTPF Rv3133C Rv2004C Rv2626C Rv2625C Rv2627C Rv2032 PANB Rv2466C Rv2035 Rv3134C Rv2962C Rv0081 Rv2630) (WHIB4 Rv2668 Rv1136 Rv3342 Rv1234 PGMA Rv3895C Rv0941C LPPJ Rv0653C Rv3479 Rv1179C Rv2478C Rv0108C Rv2184C PDXH Rv0502 CDH Rv1352 FABG3 Rv3123 Rv1453 THYA LIPH Rv1893 Rv0650 ECHA14 Rv0771 Rv1413 Rv0121C Rv3654C Rv2044C LIPF Rv2670C CPSY Rv2297 Rv0165C PKS11 Rv1362C Rv2799 Rv1363C Rv2255C Rv1931C Rv3501C VIUB APT Rv1861 SECG Rv3860 Rv0149 Rv0269C Rv2639C Rv1151C Rv0230C MOAC3 Rv2722 TRXA Rv3891C Rv0188 Rv1535 Rv2288 Rv2657C Rv3764C Rv1230C Rv3288C LPQH Rv0695 Rv3633 Rv3616C Rv3399 LPRF Rv2638 Rv3615C Rv3614C PAPA3 FRDC Rv2129C Rv1926C SODA Rv2633C Rv2557 LAT Rv3733C Rv2161C PKS4 Rv2558 Rv2632C PKS3 Rv1868 NARG Rv3751 Rv0696 SIGF Rv3679 Rv2160C RSBW Rv2253 Rv2336 FUSA2 PAPA1 Rv2598 RELA PKS2 Rv3241C Rv1639C SUHB Rv1871C Rv3500C EPHA Rv1184C Rv0387C Rv1433 OMPA Rv0171 Rv3496C CDD MOAE2 Rv2024C FADE26 SCOA Rv0657C AMT Rv2348C Rv3750C Rv3491 Rv0137C Rv3887C Rv1037C Rv3874 Rv2137C LLDD2 Rv2311 Rv2205C NARJ NARH Rv2369C Rv0621 Rv1398C FURA Rv1154C Rv2472 Rv3449 FADD16 LPQO Rv0168 Rv0767C Rv0736 INFC FRDB Rv0245 NARI Rv0167 RIBC Rv0258C Rv2765 Rv1425 Rv1968) (PDHA Rv3802C LPPD GUAB1 Rv1783 Rv1782 Rv0126 GLYS Rv1892 Rv1978 Rv0654 Rv1885C Rv0760C Rv3850 OBG FBPB PRA Rv2958C NRDE Rv1956 Rv0192 Rv1988)	Cell wall, cell processes, and metabolism

The whole-genome gene expression image (Figure 1) was divided into four zones, each annotated with major gene clusters therein and the functional classes of representative genes. The list of genes in each zone is available [see Additional file 5].

**Table 3:** The distribution (percentage %) of functional categories for genes in the major clusters of each zone on the gene expression map.

Clusters in	Vil	Lipid	Info	Cell-W-P	Ins-S-P	Meta-Res	?	Reg	Hypo
Zone-1	5	20	0	15	0	30	0	0	30
Zone-2	0	2	5	32	3	34	0	2	22
Zone-3	3	0	38	16	0	22	0	4	17
Zone-4	6	8	5	20	2	21	1	7	30

Vil: virulence, detoxification, adaptation. Lipid: lipid metabolism. Info: information pathways. Cell-W-P: cell wall and cell processes. Ins-S-P: insertion sequences and phages. Meta-Res: intermediate metabolism and respiration. ?: unknown function. Reg: regulatory function. Hypo: conserved hypothetical proteins.

**Table 4:** Transcriptional regulators associated with major gene clusters based on microarray analysis.

Gene/ORF	Gene Product	Assocated Gene Cluster
Rv2989	Probable transcriptional regulatory protein	Respiration and energy production
Rv2258c	Possible transcriptional regulatory protein	Information pathways
Rv3334	Probable transcriptional regulatory protein probably MerR-family	Cell wall, cell processes, and metabolism
Rv0465c	Probable transcriptional regulatory protein	Ditto
Rv0081	Probable transcriptional regulatory protein	Ditto
Rv3681c	Probable transcriptional regulatory protein	Ditto
Rv0653c	Possible transcriptional regulatory protein probably TetR-family	Ditto
Rv0165c	Possible transcriptional regulatory protein probably GntR-family	Ditto
Rv1931c	Probable transcriptional regulatory protein	Ditto
Rv1151c	Probable transcriptional regulatory protein	Ditto
Rv1956	Possible transcriptional regulatory protein	Ditto

**Table 5:** Conserved hypothetical proteins associated with the gene clusters of each zone on the gene expression map.

Clusters in	Conserved Hypothetical Proteins
Zone-1 (Intermediate and lipid Metabolism)	(Rv2425c Rv3541c Rv2393 Rv3701c Rv2052c Rv1728c)
Zone-2 (Energy and Respiration)	(Rv1251c Rv2054 Rv1378c Rv3321c Rv2901c Rv2949c Rv0546c Rv1870c Rv1711 Rv3672c Rv2554c Rv3856c Rv3212 Rv1043c Rv0525 Rv1339 Rv2956 Rv2759c Rv2879c Rv1780 Rv2475c Rv2752c Rv0177 Rv2367c) (Rv1632c Rv2808 Rv3122 Rv1907c)
Zone-3 (Information Pathways)	(Rv2147c Rv3267 Rv1697 Rv3909 Rv2574 Rv3376 Rv2125 Rv1546 Rv1099c Rv2908c Rv1073 Rv0636 Rv0637 Rv0277c) (Rv2826c Rv2360c Rv2827c Rv3647c Rv0299 Rv2822c Rv0057)
Zone-4 (Cell Wall, Cell Processes, and metabolism)	(Rv0516c Rv0991c Rv2020c Rv0968 Rv0967 Rv1813c Rv1847 Rv0190 Rv0080 Rv2699c Rv2629 Rv0571c Rv2623 Rv2005c Rv2004c Rv2626c Rv2627c Rv2032 Rv2466c Rv2035 Rv3134c Rv0941c Rv2478c Rv2184c Rv0502 Rv1352 Rv1893 Rv1413 Rv0121c Rv3654c Rv2044c Rv2670c Rv3860 Rv0269c Rv2722 Rv0695 Rv3633 Rv3616c Rv3399 Rv2638 Rv3615c Rv3614c Rv3733c Rv2632c Rv1868 Rv2598 Rv1871c Rv0387c Rv2024c Rv0657c Rv2137c Rv2311 Rv2205c Rv1398c Rv2472 Rv0767c Rv0258c Rv1425 Rv1978 Rv1885c Rv0760c PRA Rv0192) (Rv2628 Rv2517c Rv2662 Rv1772 Rv2016 Rv0572c Rv2630 Rv1179c Rv0108c Rv3123 Rv2297 Rv2255c Rv1535 Rv2288 Rv3288c Rv2633c Rv2557 Rv2558 Rv2336 Rv3491 Rv2369c Rv1154c Rv3850)
