# Supplementary material for: The gene expression data of Mycobacterium tuberculosis based on Affymetrix gene chips provide insight into regulatory and hypothetical genes
Source: BMC Microbiol. 2007 May 14;7:37. doi: 10.1186/1471-2180-7-37 (PMC1884158; doi:10.1186/1471-2180-7-37)
Supplement: Additional File 1 — Microarray data. The microarray data and supplementary materials produced in this study. [file 1471-2180-7-37-S1.html]

The Gene Expression Data of Mycobacterium tuberculosis
Based on Affymetrix GeneChips Provide Insight into Regulatory and Hypothetical Genes


### The Gene Expression Data of Mycobacterium tuberculosis Based on Affymetrix GeneChips Provide Insight into Regulatory and Hypothetical Genes

#### Pacific Tuberculosis and Cancer Research Organization Irvine, CA, USA

 [PubMed]
  
*Background: Tuberculosis remains a leading infectious disease with global
public health threat. Its control and management have been complicated by
multi-drug resistance and latent infection, which prompts scientists to find
new and more effective drugs. With the availability of the complete genome
sequence of the etiologic bacterium, Mycobacterium tuberculosis,
it is now feasible to search for new drug targets by sieving through
a large number of gene products and conduct genome-scale experiments
based on microarray technology. However, the full potential of genome-wide
microarray analysis in configuring interrelationships among all genes in
M. tuberculosis has yet to be realized. To date, it is only possible to assign
a function to 52% of proteins predicted in the genome.
Results: The study presented here addresses the above issue using the
high-resolution Affymetrix oligonucleotide GeneChip. Approximately one-half
of the genes in the genome were found to always express, including more than
100 predicted conserved hypotheticals. The gene expression profiles were
analyzed and visualized through cluster analysis to epitomize the full
details of genomic behavior. Broad patterns derived from genome-wide
expression experiments in this study have provided insight into the
interrelationships among genes in the basic cellular processes of M. tuberculosis.
Conclusions: Our results have confirmed several known gene clusters,
such as energy utilitzation, protein synthesis, and lipid metabolism,
and also hinted at potential roles of hypothetical and regulatory proteins.*

**[Data:]**   
**Gene expression (mean and standard deviation) of
active MTB genes under log phase growth**   

**[Microarray Data:]**   

MTB growth data-1   

MTB growth data-2   

MTB growth data-3   

MTB growth data-4   

MTB growth data-5   

MTB growth data-6   

MTB growth data-7   

MTB growth data-8   

MTB growth data-9   

MTB growth data-10   

MTB growth data-11
